# Hydroponic Common-Bean Performance under Reduced N-Supply Level and Rhizobia Application

**DOI:** 10.3390/plants12030646

**Published:** 2023-02-01

**Authors:** Ioannis Karavidas, Georgia Ntatsi, Theodora Ntanasi, Anastasia Tampakaki, Ariadni Giannopoulou, Dimitra Pantazopoulou, Leo Sabatino, Pietro P. M. Iannetta, Dimitrios Savvas

**Affiliations:** 1 Laboratory of Vegetable Production, Department of Crop Science, Agricultural University of Athens, Iera Odos 75, 11855 Athens, Greece; 2 Department of Agriculture, Hellenic Mediterranean University, Estavromenos, 71410 Heraklion, Greece; 3 Department of Agricultural, Food and Forest Sciences, University of Palermo, 90128 Palermo, Italy; 4 Ecological Sciences, James Hutton Institute, Dundee DD2 5DA, UK

**Keywords:** *Phaseolus vulgaris* L., biological nitrogen fixation, nodulation, yield, nitrogen, strain

## Abstract

This study aims to explore the possibility of a reduced application of inorganic nitrogen (N) fertiliser on the yield, yield qualities, and biological nitrogen fixation (BNF) of the hydroponic common bean (*Phaseolus vulgaris* L.), without compromising plant performance, by utilizing the inherent ability of this plant to symbiotically fix N_2_. Until the flowering stage, plants were supplied with a nutrient solution containing N-concentrations of either a, 100%, conventional standard-practice, 13.8 mM; b, 75% of the standard, 10.35 mM; or c, 50% of the standard, 6.9 mM. During the subsequent reproductive stage, inorganic-N treatments b and c were decreased to 25% of the standard, and the standard (100% level) N-application was not altered. The three different inorganic-N supply treatments were combined with two different rhizobia strains, and a control (no-inoculation) treatment, in a two-factorial experiment. The rhizobia strains applied were either the indigenous strain *Rhizobium sophoriradicis* PVTN21 or the commercially supplied *Rhizobium tropici* CIAT 899. Results showed that the 50–25% mineral-N application regime led to significant increases in nodulation, BNF, and fresh-pod yield, compared to the other treatment, with a reduced inorganic-N supply. On the other hand, the 75–25% mineral-N regime applied during the vegetative stage restricted nodulation and BNF, thus incurring significant yield losses. Both rhizobia strains stimulated nodulation and BNF. However, the BNF capacity they facilitated was suppressed as the inorganic-N input increased. In addition, strain PVTN21 was superior to CIAT 899—as 50–25% N-treated plants inoculated with the former showed a yield loss of 11%, compared to the 100%-N-treated plants. In conclusion, N-use efficiency optimises BNF, reduces mineral-N-input dependency, and therefore may reduce any consequential negative environmental consequences of mineral-N over-application.

## 1. Introduction

Nitrogen (N) is an essential macronutrient, required in high amounts by plants for optimum growth and yield [1]. The conversion of inert atmospheric N_2_ into reactive forms (NH_3_) through the industrial Haber–Bosch process helped trigger the “green evolution”, increasing crop productivity and reducing hunger, despite the increased human population. By 1950, the global population had almost tripled, while synthetic mineral-N-fertilization enabled crop production to meet almost half (48%) of the global food demand [2]. However, the increased inorganic-N application in combination with the difficulty in precisely meeting plant N-requirements, leads to severe negative environmental consequences. The N surplus is emitted to the atmosphere as ammonia, N or nitrous oxide, and leached to groundwater as nitrates—posing risks to human health, ecosystem services and biodiversity [3,4]. Today, increased environmental awareness among consumers [5] and conflict-related disruptions in the global fertilizer trade [6] has encouraged growers to adopt reduced and more sustainable mineral-N management practices.

Biological nitrogen fixation (BNF), occurring through the symbiosis of legumes with rhizobia, is proposed as an eco-friendly and sustainable source of N, compared to its industrial counterpart, as it can provide almost 50–70 Tg N per year in agricultural systems [7,8]. However, its contribution to N balance in agricultural ecosystems relies on the environmental conditions, including management, soil-microbial and physicochemical-properties, legume genotype, rhizobia species and their interactions [9]. Except for the level of N input, strategies that either foster nitrogen use efficiency (NUE) or mitigate the requirements for an external N supply are essential for the establishment of sustainable N-fertilization management [10].

Common bean (*Phaseolus vulgaris* sp.) grains or green pods are a rich source of plant protein, essential minerals, carbohydrates, fibres and several bioactive compounds [11,12,13]. In addition to their high nutritional value, the high adaptability of this crop species to different edaphoclimatic conditions [14], its capability of utilizing atmospheric N, and short growth-cycle compared to other legumes [15], render the common bean as the most popular legume for human consumption, with high socioeconomic value, especially for the developing countries [16].

The average proportion of plant N derived from air (%Ndfa) in the common bean is approximately 39%, which is considered low compared to that of other widely cultivated legumes, such as the fava bean (75%); soybean, pea and lentil (62–94%); and cowpea, chickpea and pigeon pea (54–58%) [17]. This is due to the poor BNF-capacity of the common bean, and optimal productivity relies on externally applied inorganic-N input [18]. However, a high inorganic-N application inhibits plant nodulation and BNF, further increasing the crop’s dependency on mineral-N addition, if yield is to be maintained [19,20]. Research has therefore been conducted to develop low inorganic-N-use management-strategies, based on optimising BNF without compromising the productivity of the common bean. For example, suppling 20–40 kg N ha^−1^ to plants at the early-growth stage, followed by rhizobia application prior to nodule formation and BNF onset [21,22,23,24] .In addition, preventing BNF suppression by N supply, e.g., at the flowering stage [25]. Whichever strategy is pursued, Barros et al. [26] and Soares et al. [22] agree that mineral-N supply during crop growth is essential to optimize the common-bean yield.

Overall, appropriate N fertilization of the soil-grown common bean still seems controversial, while the diverse soil temperature, pH, nutrient availability and rhizobia genotypes challenge the promotion of a standard and efficient strategy [9]. However, the controlled root-environment, the opportunity for precise delivery of fertilizer treatments, and the elimination of nutrient losses in soilless cultivation-systems offers a promising and sustainable approach to optimising common-bean yield. Despite this potential, only a small number of research studies have been conducted aiming to optimise BNF and minimise mineral-N application to the common bean grown in soilless systems [27]. A range of mineral-N application levels, in combination with rhizobia inoculation in the hydroponically cultivated common bean was evaluated by Kontopoulou et al. [28] and Arcas-Pilz et al. [29]. However, given the severe N starvation applied in both studies, BNF capacity was too low to meet the plant requirements, and so yield and profitability were compromised. The common bean has also been grown hydroponically by Kouki et al. [30], Jiang et al. [31], Pradham et al. [32] and Perez et al. [33]. However, these studies were based on short-term experiments which evaluated the impact of different rhizobia species and/or N supply mainly on nodulation and BNF of the common bean during the early developmental stages of the plant. In addition, Franco and Munns [34] recommended gravel as a substrate for soilless cultivation, since the common bean exhibits poor nodulation when grown exclusively in solution culture.

Given this background, this study aims to identify a strategy for a soilless hydroponically cultivated common-bean, whereby BNF is optimised and inorganic-N use is minimised such that yield is not compromised. For this purpose, the common bean was cultivated hydroponically and exposed to varying inorganic-N application-regimes schemes during the different developmental stages. Considering the beneficial effects of N on nodulation and plant growth and the importance of advanced N-fixing activity during flowering on the subsequent reproductive stage [35] a two-phase mineral-N treatment was therefore imposed in addition to the conventional standard levels, with either 75 or 50% of the standard-practice quantity (100%) applied during the vegetative stage, which was then reduced to 25% of the standard from flowering to the end of the cropping period. Control plants were supplied with nutrient solution (NS) comprising a conventional-standard (100%) quantity of inorganic-N for common bean for their whole growth cycle. In addition to the different inorganic-N application regimes, the potential benefits of the different rhizobia genotypes to BNF was also tested, using either the indigenous strain *Rhizobium sophoriradicis* PVTN21 or *Rhizobium tropici* CIAT 899, with non-inoculated plants serving as the control treatment.

## 2. Results

As shown in Table 1, the different inorganic-N application treatments did not affect the common-bean-shoot fresh biomass significantly, during either the vegetative- or flowering-stages. However, during first-pod emergence, the shoot fresh-weight was reduced by 9% when the inorganic-N application was reduced from 50% to 25%, compared to that of plants with the conventional full (100%) inorganic-N applied. Furthermore, the inoculation of the common bean with rhizobia and the strain used had no significant impact on common-bean growth, regardless of the crop developmental stage.

In contrast, nodulation was significantly affected by both the level of mineral-N applied and rhizobial inoculation (Table 1). Decreased inorganic-N supply at the early- and vegetative-growth stages at 75% or 50% of the standard level, significantly increased the nodule number per plant, compared to 100% mineral-N application, when the plants were inoculated with either of the two rhizobia strains tested. However, the number of nodules per plant at the flowering stage and prior to first-pod emergence was significantly greater when inorganic-N was at the initial-growth stage, i.e., 50%, compared to 75%, of the control treatment i.e., 100% N. Nodulation was enhanced during the vegetative- and the flowering-stages for plant inoculated with strain PVTN21, irrespective of the N supply level, while prior to pod formation the difference was significant only when the N supply during the early growth stage was reduced, irrespective of the reduction level (75% or 50%). However, the strain CIAT 899 increased the nodulation of the common bean to significantly higher levels only when the N supply at the early-growth stage was reduced by 50% at the vegetative stage and by both 75% and 50% at the flowering stage. Furthermore, prior to first-pod emergence, the inoculation with CIAT 899 had no impact on the number of nodules per plant.

**Figure 1:**
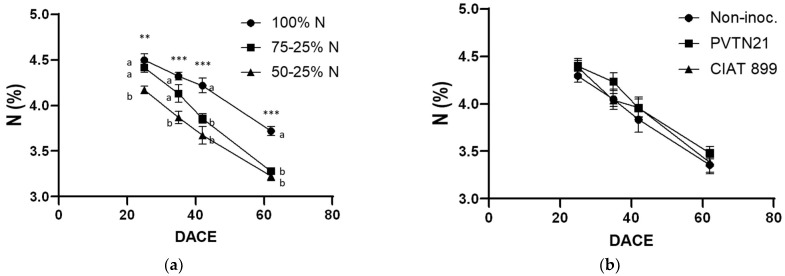
Total-N content in the aboveground biomass (shoots) of common-bean (*Phaseolus vulgaris* L.) plants during vegetative-, flowering-, just-prior-to-pod-formation, and reproductive-growth stages (25, 35, 42, and 62 DACE, respectively). The graphs show: (**a**) the impact of different mineral-N treatments; and (**b**) inoculation with rhizobia. *** and ** denotes statistical significance according to Duncan’s multiple range test at *p* < 0.001 and *p* < 0.01, respectively.

As shown in Figure 1a, the reduction of the inorganic-N supply to the common-bean plants reduced the total-N content of the shoot compared to that measured in plants supplied with the standard (100%) level throughout the cropping period. Thus, plants receiving 50% of the standard N supply during the early-growth stage exhibited a significantly lower shoot-N-content at all sampling dates, compared to 100% N supply. However, those treated with 75% of the standard N supply during the early-growth stage exhibited a significant decrease in the shoot total-N only on the last two sampling-dates (after day 40 from crop establishment), particularly when the N supply was reduced to 25% of the standard level. Overall, the shoot N-content in all treatments declined gradually with time, and during the reproductive stage it ranged below 4% and 3.5% in the plants grown under adequate (100%) and deficit N-supply, respectively. Inoculation of the common bean with either PVTN21 or CIAT 899 strains did not affect the shoot N-content of the host legume during the whole cultivation period (Figure 1b). Finally, the N-supply regime and the inoculation with rhizobia did not reveal any interaction concerning the N content of the common bean.

According to Table 2, neither Δ nor C (%) were significantly influenced by either the inorganic-N or rhizobial inoculation-treatments at the early stages of pod formation. However, at the same developmental stage, with treatments with a restricted inorganic-N supply (75–25 and 50–25%) shoots showed an increased C:N ratio, and this difference was also recorded for the following reproductive stage. At the reproductive stage, regardless of the different inorganic-N treatments, shoots of plants subject to strain PVTΝ21 showed a significantly decreased C:N ratio. Additionally, the interaction between these two experimental variables showed that application of strain PVTN21 increased shoot C:N ratio under the lowest N-supply treatment (50–25%) at both vegetative and reproductive developmental-stages. Moreover, by the reproductive stage, both reduced inorganic-N treatments had significantly reduced the shoot Δ values of the common bean in the shoot dry-biomass.

The shoot-N percentage derived from the atmosphere (%Ndfa) was proportionally increased at both sampling dates as the N-supply level at the early-growth stage was reduced (Table 3). However, the total amount of biologically fixed N (BNF) was differently influenced by the N-supply level on the two sampling dates. Thus, at the early-pod-development stage, the BNF was equally increased by the two treatments with restricted N-supply, compared to standard N-supply. In contrast, at the reproductive stage, BNF in the 75–25% treatment was significantly lower than in the full, standard mineral-N supply (100%). Only the 50–25% mineral-N treatment increased the BNF, compared to 100% of the standard amount. Nevertheless, a clearly beneficial effect of inoculating the common bean with rhizobia on the %Ndfa and total fixed-N (BNF) appeared only when the inoculation with the PVTN21 strain was combined with a reduction of the N supply during the early-growth stage to 50% of the standard.

With respect to yield, reducing the quantity of mineral-N applied, decreased fresh-pod production. Plants grown under 50–25% and 75–25% mineral-N regimes produced fewer pods (19.7 and 39.3%, respectively, while the mean weight of the harvested pods was also restricted by 3.4 and 7.4%, respectively. The adverse effects of reduced inorganic-N supply on both yield components resulted in total yield losses (g plant^−1^) of 22.5 and 43.8%, respectively. However, rhizobia inoculation showed increased average-pod-weight compared to non-inoculated plants (Table 4). In addition, inoculation with strains PVTN21 or CIAT 899 significantly increased pod number per plant, by 13.1 and 6.9%, respectively. Moreover, PVTN21 and CIAT 899 strains enhanced the total fresh common-bean-pod yield per plant by 19.5 and 9.2%, respectively, compared to no inoculation.

In addition, the impact of inoculation was different for each N-supply treatment. At full N-supply (100% of the standard) inoculation, regardless of the strain applied, had no impact on total pod yield, or on the two yield components. Under the 75–25% N treatment, both rhizobia strains significantly increased total pod yield and the yield components, compared to no inoculation. However, under the 50–25% mineral-N treatment, PVTN21 allowed significantly greater total-pod-yields than CIAT 899.

## 3. Discussion

During early developmental stages, 15–20 days after emergence, common-bean growth relies exclusively on external-N inputs because of the lack of synchronization between BNF onset and the N depletion in the cotyledons [36]. Thus, the ideal inorganic-N fertigation strategy to optimise growth and yield seems controversial for a pulse crop, as applying a sufficient mineral-N supply to stimulate growth may also discourage root nodulation and BNF [34,37]. Contrary to the well-documented inverse relation between N availability and nodulation [19,22,25,31], Kontopoulou et al. [28] found that severe N-starvation at this initial-growth stage hinders rather than benefits bean nodulation, because the N deficiency limits the energy input from photosynthates. In the same study, N input up to 33% of plant N-requirements benefited root nodulation but restricted plant growth and, concomitantly, the pod yield. In the present study, the supply of a nutrient solution (NS) sufficient to meet 50% of plant N-requirements during the early plant-developmental-stage (to early flowering) and 25% thereafter, promoted nodulation without affecting the growth rate of the plants, until the early-pod-formation stage. According to Zoffoli et al. [38], N supply in combination with *Rhizobium tropici* CIAT 899 during the early-developmental stage, when BNF is not yet established, accelerated plant growth without inhibiting nodulation. However, further inorganic-N supply until the late-vegetative stage did not benefit common-bean growth, and eventually compromised root nodulation. This impact was also verified in this study, as neither inorganic-N supply nor rhizobia inoculation affected the common-bean biomass. Additionally, N supply above 50% of the plant N-requirements did not benefit vegetative growth, and inhibited root nodulation by rhizobia. However, the rhizobium strains CIAT 899 and PVTN21 enhanced nodulation only when the mineral-N supply was restricted. The suppression of root nodulation by rhizobia under standard quantities of mineral-N application has been reported previously by several researchers, including Rebeschini et al. [39], Głodowska et al. [40] and Souza et al. [16]. Irrespective of the level of N input, the native strain PVTN21 enhanced nodulation to a greater extent than the commercial rhizobia strain CIAT 899, the effectiveness of which weakened as cultivation progressed. Efficient symbiotic interaction between the common bean and native rhizobia compared to rhizobia CIAT 899 was also recorded, in the study by Tajini et al. [41], highlighting the importance of rhizobia Xstrain interaction for common-bean nodulation.

The application of an NS containing inorganic-N levels at 75% of the standard N requirements during the early-growth stage did not restrict the shoot % N content compared with the full (100%) quantity at the same plant-developmental stage. However, during the reproductive stage, when the inorganic-N supply was reduced to 25%, the shoot % N content was significantly reduced, compared to the 100% level. These results indicate that N acquired via BNF in the 75–25% treatment could not compensate for the missing 75% of the standard N-requirement during the reproductive-growth stage. Moreover, the increased nodulation of the plants grown under 75% N during the early-vegetative stage signifies that the application of 10.35 mmol/L N through the NS during the flowering stage was beyond the plant N requirements. Moreover, the N concentration of the plants grown under 100% N decreased as the cultivation progressed. In contrast, according to the studies of Westerman et al. [42], Araújo et al. [43], George and Sigleton [44] and Soratto et al. [45], the flowering- and especially the reproductive-stage are considered as the most N-demanding developmental stages of common-bean plants. Overall, given that the total N input is the sum of both supplied and fixed N, which are closely interrelated, it is difficult to achieve an optimum supply of inorganic-N that addresses the timing of the N-demand of the hydroponic common-bean, which is also dependent on cultivar, rhizobia strain, and prevailing environmental conditions [27].

Carbon isotope composition (δ^13^C) serves as either a long-term transpiration rate or a water and/or nitrogen use-efficiency (NUE) indicator [46,47,48,49]. According to Fu et al. [50], *Phaseolus vulgaris* sp. recorded lower Δ values under sufficient soil N availability combined with a limited water supply, an outcome that was mainly ascribed to the reduced photosynthetic capacity of plants under N stress. However, Knight et al. [51] observed a positive relationship between Δ and BNF activity of the common bean. Additionally, δ^13^C of the common bean was not influenced by the different fertilization-regimes in the study by Smith et al. [52]. In contrast, in the paper by Jiang et al. [31], in which the common bean was supplied with an NS under various mineral-N levels, as the N supply decreased, the δ^13^C became less negative, and thus the Δ values decreased, while a positive correlation between δ^13^C and %Ndfa was also observed. These findings are partially in agreement with the present study, in which the Δ values were mainly influenced by external N inputs rather than by BNF.

Except for the Δ values, the C:N ratio is also considered an NUE indicator, according to Wang et al. [53], and is highly affected by fertilization management. In particular, an elevated C:N ratio demonstrates enhanced NUE in plants [54]. Additionally, Sun et al. [55] reported that an increased inorganic-N supply enhances both the C and N content in plant shoots, but decreases the C:N ratio. In the current study, the different inorganic-N fertigation treatments did not affect the total C content of the aboveground biomass, and thus the variations observed in C:N are mainly ascribed to the differences in the shoot N content. In particular, both inorganic-N deficit treatments elevated the C:N ratio in the aboveground biomass during both early-pod-formation and late-reproductive stages. It therefore seems that the common-bean plants exhibited a higher NUE under a suppressed inorganic-N supply. These results further confirm the suggestion that reducing the inorganic-N supply to the common bean could not be compensated for by the increase in nodulation and BNF, and that it finally restricted the total N available to the plants. Similar reports concerning the relation between inorganic-N input and C:N ratio were also found in the study by Kontopoulou et al. [56], in which the limited N-availability in an organic common-bean crop increased the shoot C:N ratio compared to that found in plants grown conventionally. In contrast, Karavidas et al. [57] found that although the organic farming practices restricted the soil N availability, the N content and the C:N ratio in common bean shoots were not influenced. This discrepancy was presumably because the shortage of soil N in the study by Karavidas et al. [57] was less restrictive than those of the current study, and those of Kontopoulou et al. [56]. Furthermore, under the 50–25% inorganic-N regime, plants that were inoculated with the strain PVTN21 exhibited a lower C:N ratio compared to the plants that were either inoculated with CIAT 899, or non-inoculated. This result might be associated with the higher BNF capacity afforded by the strain PVTN21, indicating that an increase in the %Ndfa in plant tissues may be associated with a reduction in the C:N ratio.

The inhibitory effects of standard levels of inorganic-N supply on BNF of the common bean found in this study are in accordance with many previous studies e.g., [19,20,31]. Moreover, the recorded %Ndfa values under either standard (100%) or deficit inorganic-N regimes here are in accordance with those recorded by Reinprecht et al. [19], who cultivated different climbing-common-bean genotypes under similar inorganic-N regimes. In particular, during the stage of first-pod emergence, as the level of N supply decreased, the N_2_ fixing-activity of the common bean increased. However, this inverse relationship was not observed during the reproductive stage. Specifically, although the plants in the two N-deficit treatments (75–25%N and 50–25% N) received only 25% of their total N needs during that stage, the common-bean plants supplied with 50% N before the flowering stage recorded higher Ndfa(%) (37%) compared to those measured in plants supplied with 75% N. Therefore, the increased BNF of plants subjected to 50% of the standard allowed for further fixed-N gains during the N-demanding reproductive stage. Moreover, Pena-Gabriales et al. [35] suggested that, to maximize BNF activity in the common bean during the reproductive stage, practices that optimize the N_2_ fixation rates during the flowering and the early-reproductive stages should be adopted. According to Muller et al. [25], elevated inorganic-N supply at the late vegetative-flowering stage inhibits nodulation and BNF of the common bean. In addition, Mastrodomenico et al. [58] concluded that the inhibitory effects of stress on nodulation and BNF during soybean flowering can be reversed if favourable growth conditions are established during the following reproductive stage. However, in the current study, decreasing the supply of mineral-N from 75 to 25% during the reproductive stage did not boost BNF to levels witnessed for plants treated with 50–25%. This outcome could be attributed to the competition of nodules and pods for photosynthates during the reproductive stage [15]. Nevertheless, this competition for photosynthates was presumably milder in plants receiving 100% of their N needs as inorganic-N, and this presumably resulted in higher total amounts of symbiotically fixed N, compared to the 75–25%-N-treated plants, despite the lower number of nodules per plant.

In a similar way to its impact on nodulation capacity, adequate N supply can be detrimental also on rhizobia efficiency in terms of N fixation in the common bean. Aouani et al. [59] emphasized the importance of inorganic-N fertilization on plant–rhizobia interactions, concluding that sub-standard N-fertigation management should be adopted to establish an efficient symbiotic process. In the present study, the BNF of plants grown under the lowest total-N-input (50–25%) and inoculated with PVTN21 was almost double compared to that of the non-inoculated plants. In addition, higher BNF was also recorded in plants inoculated with CIAT 899 compared to non-inoculated treatment, though this is ascribed to the higher plant dry-biomass rather than higher %Ndfa per se, which did not differ from that of non-inoculated plants. Several studies [60,61,62,63] report the broad-host-range rhizobia-strain CIAT 899 as a suitable inoculant for *Phaseolus vulgaris sp*., while other studies [64,65,66] highlight the potential of native rhizobia for enhanced BNF in the common bean. In the present study, the common bean was cultivated hydroponically on a chemically inert growing-medium (perlite), and thus the soil physicochemical properties and the presence of indigenous soil-borne rhizobia did not interfere with the inherent BNF-potential of the tested strains. Consequently, the reduced %Ndfa and BNF of plants inoculated with *Rhizobium tropici* CIAT 899 indicate that this strain is a poor inoculant, while PVTN21 can be considered a much more efficient strain in terms of BNF, at least for the common-bean genotype cultivated in the current study.

Although plants treated with 75–25% N and the 50–25% inorganic-N regime exhibited similar NUEs, the 50–25% N treatment resulted in a 38% higher total fresh-pod yield per plant, compared to the 75–25%, which was mainly ascribed to the increased amount of %Ndfa, by approximately 60%. In addition, the enhanced N-fixation in the 50–25% N treatment reduced by 25% the yield gap between the plants treated with reduced or adequate N, compared to the 75–25% treatment. This finding highlights the importance of the BNF as a significant determinant of the common bean yield-stability when the input of inorganic-N is restricted. The potential of fixed N_2_ to meet crop N-demand and avoid yield losses was also reported in the study of Pacheco et al. [67]. Loses in fresh-pod yield under inadequate inorganic-N supply is a result of the reduced number and size of pods per plant. However, the most significant difference between treatments was recorded for pod number per plant. According to Fageria and Santos [68], the number of pods per plant is the most significant determinant of total yield for the common bean, and this attribute is very sensitive too when the common bean is cultivated under less-than-favourable growth conditions [69]. Moreover, common-bean plants inoculated with the strain PVTN21 showed the highest BNF and an enhanced NUE. These synergistic effects boosted the yield of the common bean in the 50–25% treatment, thereby restricting the yield reduction to only 11.2%, compared to that harvested from plants treated with 100% inorganic-N. This was despite the strong reduction in the inorganic-N supply of the 50–25% regime—a 64% savings in inorganic -N for the whole cultivation period. Additionally, the commercial strain CIAT 899 also enhanced the total yield by 21% under 50–25% N, compared to non-inoculation. Beneficial effects of *R. tropici* CIAT 899 on common-bean yield under suppressed N-availability were also observed in the work of Barros et al. [26] and Ndakidemi et al. [70], while Cardillo et al. [71] reported that CIAT 899 did not influence common-bean productivity. due to the presence of a sufficient population of native soil-rhizobia.

## 4. Materials and Methods

### 4.1. Plant Material, Experimental Conditions and Rhizobia Strains 

The climbing common bean (*Phaseolus vulgaris* cv. Borloto) was cultivated hydroponically under different levels of inorganic-N applications during October–December 2020 at the experimental facilities of the Laboratory of Vegetable Production in the Agricultural University of Athens (37°58′56.4′′ N 23°42′15.7′′ E). During the vegetative stage (seedling to flowering stages) the common-bean plants were supplied with a nutrient solution (NS) containing levels of inorganic-N fertiliser at either a) the full conventional-standard amount (100%) according to the standard practices; or b) 75 and c) 50% of this standard, respectively. During the reproductive stage, the inorganic-N levels in the nutrient solution (NS) supplied to the plants in treatments (b) and (c) were further decreased to 25% of the standard plant-N requirements. The three different inorganic-N application treatments were combined with rhizobia-inoculation treatments comprising either the commercial strain *Rhizonium tropici* CIAT 899 or *Rhizobium sophoriradicis* PVTN21, a Greek indigenous strain originating from Tinos Island [72], or a non-inoculated (control) treatment, using a 3 × 3 factorial experimental-design. Each of the nine treatments obtained by the combination of these two experimental factors was replicated 6 times.

The plants were cultivated in an open hydroponic system which was accommodated in a glasshouse and comprised 9 independent circuits. Each N-supply treatment was replicated three times (3 circuits), while each circuit accommodated all three rhizobia-inoculation treatments. Perlite placed in bags (33 L) was used as substrate and each hydroponic circuit accommodated 6 bags. To avoid bacteria leaching from one bag to another within the same circuit, each of the nine treatments was accommodated in one of the nine circuits, while each perlite bag served as a replication.

### 4.2. Seed Germination, and Plant-Inoculation Process 

Common-bean seeds were germinated in a temperature-controlled (23–26 °C) incubation chamber. After 4 days, the seeds that were germinated were either inoculated with one of the two tested rhizobia species, or non-inoculated. Subsequently, the seeds were transferred (10 February 2020) to perlite bags saturated with an NS suitable for bean [28], with 6 seeds per bag. During the first two days after sowing, no NS was supplied to the germinated seeds, to prevent bacteria leaching, which could potentially contribute to inoculation failure. Then, the perlite bags were slit at the bottom to allow for free drainage of the excess NS [73]. No pests and diseases occurred during the whole cultivation period, and thus no plant-protection products were applied.

The common bean was inoculated with either the commercial strain CIAT 899 or the indigenous strain PVTΝ21, originating from the Greek island Tinos and isolated by the Laboratory of General and Agricultural Microbiology of AUA [73]. During inoculation, germinated common-bean seeds were placed in a liquid culture (10^9^ cfu mL^−1^) of one of the above rhizobia strains for 10 min, and then the inoculated seeds were planted into the well-saturated perlite bags. In addition, to guarantee successful bean-inoculation, seven days after sowing, the common-bean seedlings were watered after the last irrigation dose of the day with a liquid culture (10^8^ cfu mL^−1^) of the respective rhizobia, at a rate of 10 mL plant^−1^. 

### 4.3. Nutrient-Solution Application

The NS applied to the plants in the different N-fertigation schemes (a, b, c) from crop establishment (2 October 2020) to flowering stage (6 November 2020) contained 13.8 mM N (12.6 mM NO_3_-N + 1.2 mM NH_4_-N), 10.35 mM N (9.15 mM NO_3_-N + 1.2 mM NH_4_-N) or 6.9 mM N (5.7 mM NO_3_-N + 1.2 mM NH_4_-N), which covered 100%, 75%, or 50% of the standard N-requirements of the common bean, respectively. The reduced anion-supply due to the reduced NO_3_-N supply in the reduced-N NS treatments (75% or 50%) was compensated for by equivalent elevation of the SO_4_^−^ and Cl^-^ concentrations. In addition, during the post-flowering and reproductive stage (7 November–22 December 2020), the N levels in the NS applied to the two N-deficit treatments were further decreased to 25% (3.135 mM NO_3_−N + 0.315 mM NH_4_-N) of the plant N-requirements. This further reduction of the NO_3_-N concentration in the NS with 25% N was compensated for by an equivalent reduction in the Ca^2+^ supply. This was opted for because the common bean is susceptible to high Cl^-^ levels in the root zone [74], while the reduction in Ca^2+^ in the NS promotes the nitrogen-fixing activity of the common bean when grown under deficit-N conditions [75]. Moreover, in soil-grown crops, the reduced N-supply is often accompanied by reduced Ca^2+^ supply, since the most common inorganic (nitrate) N fertilizer is calcium ammonium nitrate [76]. Furthermore, due to an adverse increase in pH (pH > 7.5) of the drainage solution on 11/20/2020, the chemical composition of the NS was further modified by altering the NO_3_-N: NH_4_-N ratio (100% N: 12 mM NO_3_−N + 1.8 mM NH_4_−N, 25% N: 2.3 mM NO_3_−N, 1.15 mM NH_4_−N), a standard practice regulating the pH in the plant-root zone in hydroponic cultivation systems [77,78]. Considering the reduced N inputs and the days that they were applied, during the whole cultivation period 53% and 64% external N inputs were conserved in the treatments 75–25% and 50–25% N, respectively. The chemical composition of the different NS applied during the whole cultivation period is shown in Table 5. The chemical composition of the irrigation water and the fertilizers used for the NS preparation are presented in Table S1 and Table S2.

The NS was supplied to common-bean plants at a rate of 2 L h^−1^ with the use of a drip irrigation system. The irrigation frequency was adapted to the plant developmental stage and was controlled using a solar meter (ALAGRO SA, Athens, Greece) to achieve a drainage fraction of approximately 30% of the total supplied NS. The percentage of the drainage solution, the electrical conductivity (EC) and the pH values were recorded daily, and used for the adjustment of the amount and chemical composition of the applied NS, if necessary. 

### 4.4. Samplings and Methods

#### 4.4.1. Growth and Yield Parameters

Three common-bean plants per treatment were collected: (A) at the vegetative-preflowering developmental stage, i.e., 25 days after crop establishment (DACE); (B) at the flowering stage (35 DACE); (C) at the post-flowering stage, i.e., during the first-pod emergence at the early development of the first pods (42 DACE); and (D) at the full-reproductive stage, i.e., at the peak of harvesting (62 DACE). The harvested plants were divided into roots, shoots, and pods when formed. 

The roots were used only for the estimation of the nodule number per plant, by counting the number of nodules per root, while the shoot samples selected on the C sampling-date included the whole aboveground biomass (shoot + early developed pods). After harvesting of the sample plants, the fresh weight of shoot was recorded immediately, and the fresh plant tissues were oven dried for 5 days at 65 °C for dry-biomass determination. After the drying of the samples, the plants collected on the D sampling-date were merged with the already oven-dried harvested pods from the same plant. The dried samples were ground through the use of a ball mill and the dried material was passed through a sieve (0.5 mm). 

Common-bean pods were harvested when they reached the marketable size and the harvest was repeated weekly. The yield performance of the hydroponic common-bean crop was evaluated by the calculation of the fresh-pod weight (g plant^−1^), the number of pods (N plant^−1^), and the mean fresh-weight (g pod^−1^).

### 4.5. Total N, Total C, Δ and BNF-Activity

Total-N determination in the powdered-plant material of the samplings A-D was conducted by applying the Kjeldhal method [79] (Kjeltec™ 8200 Auto Distillation unit). In addition to total-N, the plant material obtained from the samplings (C) and (D) was further analysed to assess total-C, ^13^C/^12^C and ^15^N/^14^N isotopes using a PDZ Europa ANCA-GSL elemental analyser interfaced to a PDZ Europa 20–20 isotope ratio mass spectrometer (Sercon Ltd., Cheshire, UK). The above measurements were conducted in the Stable Isotope Facility of UC-Davis, CA, USA. The δ^13^C and δ^15^Ν values are expressed following the international standards VPDB (Vienna Pee Dee Belemnite) and Air for carbon and nitrogen, respectively [80]. The δ^13^C values were used for Δ determination (1) which served as a photosynthetic-rate indicator [46], while the δ^15^N values were employed for the estimation of the biological fixing activity (BNF) of the common-bean plants based on the natural abundance method of isotope ^15^N. The Δ values were calculated using the following equation, as described by Fu et al. [50]:

(1) $$\Delta =\frac{\delta \alpha -\delta p}{1+\delta p}$$

where δp is the δ^13^C_PDB_ value of the plant and δa is the δ^13^C_PDB_ value of the atmospheric CO_2_ (−8‰). The proportion of N derived from the atmosphere (%Ndfa) in the plant tissues was calculated through the use of the δ^15^N values of the tested legume (common bean) and the reference plant, which was a non-legume plant that was cultivated in the same soil at the same growth stage, through the following equation [81]:

(2) $$\mathrm{Ndfa}\left(\%\right)=\frac{\delta 15Ν\mathrm{reference}\;\mathrm{plant}-\delta 15\mathrm{Nlegume}}{\delta 15\mathrm{Nreference}\;\mathrm{plant}-B}\times 100\;$$

where the ‘B’ value is δ15Ν (‰) in the shoot of the respective legume grown on an inert medium and starved of N throughout its life, thereby being fully dependent upon N_2_ fixation. In the current study, the B value used was (−2.16) for common-bean shoots, as suggested by Unkovich et al. [81]. The reference plant used for the determination of the %Ndfa in the common bean was *Citrullus lanatus* cv. MiniMax F1, which was also exposed to the different N-fertigation schemes and served as a reference plant for each of the N-supply treatments. The total amount of N_2_ derived from the atmosphere in the aboveground dry biomass (DM) of the common bean (BNF, kg ha^−1^) was estimated using the following equation [82]:

(3) $$\mathrm{BNF}\;\left(N\frac{\mathrm{kg}}{\mathrm{ha}}\right)=\left(N\left(\%\right)\times \;\mathrm{DB}\;\left(\frac{\mathrm{kg}}{\mathrm{ha}}\right)\times \;\mathrm{Ndfa}\left(\%\right)\right)/100$$

### 4.6. Statistical Analysis

To investigate the main effects of the different N-fertigation schemes (Factor 1) and the inoculation of the common bean with rhizobia (Factor 2), a two-factorial ANOVA analysis was applied. When the two-way ANOVA analysis was significant at *p* ≤ 0.05 level, the treatment means were separated by applying Duncan’s multiple range test. The statistical analysis was conducted using the STATISTICA 12.0 software package (StatSoft Inc., Tulsa, OK, USA).

## 5. Conclusions

This study supports the hypothesis that inoculation of the common bean with efficient rhizobia strains such as *Rhizobium sophoriradicis* PVTN21 when growing in soilless systems allows for a substantial reduction in the inorganic-N input, through the nutrient solution (NS) with minimal yield-losses. In the current study, a reduction of 64% in the inorganic-N-supply via the NS in the 50–25% N treatment incurred a pod yield-loss of only 11.2%, compared to full coverage of the plant needs with synthetic N-fertilizers when the seeds were inoculated with PVTN21 prior to sowing. In contrast, the same N-regime without inoculation with PVTN21 incurred yield losses of 35% compared to the 100% N-standard. Consequently, NUE (C:N ratio) increased by 9.8% under the combined 50–25%-inorganic-N and strain-PVTN21 treatments. The positive effect of inoculation with PVTN21 upon yield was due to a synergistic effect of increased %Ndfa, due to sub-standard mineral-N supply (50–25%) and the inoculation with PVTN21 (compared to the 100% N-standard and no inoculation). The small yield-losses under the 50–25% inorganic-N supply may be due to the inherent low BNF-capacity of currently available common-bean genotypes, despite the variety tested here showing an almost doubling of nodulation (with PVTN21). In addition, the current study highlights the fact that the commercial strain CIAT 899 was inferior to the native stain PVTN21 in terms of nodulation and BNF capacity, at least for the cultivar ‘Borloto’.

## Figures and Tables

**Table 1:** Impact of different inorganic-N fertigation schemes and rhizobia inoculants on the growth and nodulation capacity of the common bean (*Phaseolus vulgaris* L.).

Treatments		Vegetative Stage		Flowering Stage		First-Pod Emergence	
N	Strain	Shoot FW (g)	Nodules (N° Plant^−1^)	Shoot FW (g)	Nodules (N° Plant^−1^)	Shoot FW (g)	Nodules (N° Plant^−1^)
Main effects							
100%		65.1	18.4	102.8	30.7	148.8 a	23.4
75–25%		59.5	30.6	96.4	55.2	141.3 ab	62.7
50–25%		68.1	73.6	104.4	89.6	135.3 b	97.9
	No inoc.	63.4	4.9	94.3	11.2	136.5	22.8
	PVTN21	64.8	87.4	110.1	105.1	146.7	107.7
	CIAT 899	64.5	30.2	99.2	59.1	142.2	47.9
Interactions							
100%	Non inoc.	69.4	0 d	108.2	8 c	145.7	13 d
	PVTN21	64.2	46 bc	111.0	64.3 b	149.5	40 d
	CIAT 899	61.6	9.3 cd	89.2	26 c	151.3	17.3 d
75–25%	Non inoc.	55.2	0 d	87.0	12.7 c	133.8	22.3 d
	PVTN21	59.3	71.7 b	99.1	91.7 b	142.4	115.7 b
	CIAT 900	64.1	20 cd	103.0	61.3 b	147.6	50 cd
50–25%	Non inoc.	65.8	14.7 cd	87.7	19.3 c	130.2	39.7 cd
	PVTN21	70.9	144.7 a	120.3	159.3 a	148.2	167.3 a
	CIAT 901	67.6	61.3 b	105.4	90 b	127.6	86.7 bc
Statistical Significance							
Nitrogen		ns	***	ns	***	*	***
Strain		ns	***	ns	***	ns	***
Nitrogen*Strain		ns	*	ns	*	ns	*

Mean values (*n* = 6) followed by different letters within the same column indicate significant differences according to Duncan’s multiple range test (*p* < 0.05): *** and * significant at *p* < 0.001 and *p* < 0.05, respectively; ns = not significant. FW denotes fresh weight.

**Table 2:** Impact of mineral-N treatments and rhizobial inoculation on Δ (‰), %C, and C:N ratio of common-bean (*Phaseolus vulgaris* L.) shoots at different growth stages.

Treatments		First Pod Emergence			Reproductive Stage		
Nitrogen	Strain	Δ	Total-C	C:N Ratio	Δ	Total-C	C:N Ratio
Main effects							
100%		22.01	39.38	9.36	21.45 a	39.38	10.60
75–25%		21.72	39.88	10.35	20.82 b	39.63	12.09
50–25%		21.58	39.54	10.84	20.72 b	39.84	12.38
	Non inoc.	21.86	39.65	10.45	20.88	39.67	11.87
	PVΤΝ21	21.61	39.41	10.01	21.00	39.59	11.39
	CIAT 899	21.84	39.74	10.09	21.10	39.59	11.82
Interactions							
100%	Non inoc.	22.30	39.25	9.31 d	21.35	39.52	10.94 de
	PVΤΝ21	21.74	39.38	9.42 cd	21.38	39.47	10.60 e
	CIAT 899	21.97	39.52	9.35 d	21.61	39.14	10.26 e
75–25%	Non inoc.	21.88	39.69	10.18 bcd	20.70	39.41	11.97 bc
	PVΤΝ21	21.49	39.84	10.65 bc	20.86	39.69	11.94 bc
	CIAT 899	21.79	40.13	10.23 bcd	20.90	39.80	12.37 abc
50–25%	Non inoc.	21.40	40.02	11.85 a	20.60	40.08	12.69 ab
	PVΤΝ21	21.58	39.03	9.98 bcd	20.76	39.60	11.64 cd
	CIAT 899	21.77	39.57	10.68 b	20.80	39.82	12.83 a
Statistical Significance							
Nitrogen		ns	ns	***	***	ns	***
Strain		ns	ns	ns	ns	ns	*
Nitrogen * Strain		ns	ns	*	ns	ns	*

Mean values (*n* = 6) followed by different letters within the same column indicate significant differences according to Duncan’s multiple range test (*p* < 0.05): *** and * significant at *p* < 0.001 and *p* < 0.05, respectively; ns = not significant.

**Table 3:** The proportion of N derived from air (%Ndfa), dry biomass (DB kg/ha) and total amount of fixed N (BNF kg/ha) in the common-bean (*Phaseolus vulgaris* L.) shoots treated with varying quantities of inorganic-N (100%, 75–25, or 50–25%), and/or inoculation with rhizobia strains (PVTN21 or CIAT899), at the different (later) growth stages of first-pod emergence and reproductive (pod formation).

Treatments		First-Pod Emergence			Reproductive		
Nitrogen	Strain	%Ndfa	DB	BNF	%Ndfa	DB	BNF
Main effects							
100%		10.36 c	759	3.35 b	24.43	2269	20.56
75–25%		15.01 b	782	4.52 a	28.16	1595	14.74
50–25%		18.23 a	755	5.15 a	38.60	1852	23.51
	Non inoc.	13.21 b	737	3.72 b	28.21	1826	16.81
	PVTN21	16.79 a	790	5.25 a	33.31	1997	23.28
	CIAT 899	13.59 b	768	4.04 b	29.67	1893	18.71
Interactions							
100%	Non inoc.	10.60	737	3.41	22.66 e	2444 a	20.02 bc
	PVTN21	11.49	756	3.64	27.37 de	2223 ab	22.61 b
	CIAT 899	8.99	786	2.99	23.27 de	2141 bc	19.05 bcd
75–25%	Non inoc.	14.94	732	4.21	28.77 cd	1458 e	13.91 d
	PVTN21	15.98	819	4.96	27.12 de	1716 de	15.48 cd
	CIAT 899	14.10	793	4.39	28.59 cd	1610 e	14.82 cd
50–25%	Non inoc.	14.11	744	3.54	33.18 bc	1575 e	16.51 cd
	PVTN21	22.88	796	7.15	45.44 a	2053 bc	31.74 a
	CIAT 899	17.69	725	4.75	37.16 b	1928 cd	22.23 b
Statistical Significance							
Nitrogen		***	ns	**	***	***	***
Strain		*	ns	*	**	*	***
Nitrogen*Strain		ns	ns	ns	*	**	**

Mean values (*n* = 6) followed by different letters within the same column indicate significant differences according to Duncan’s multiple range test (*p* < 0.05): ***, **, * significant at *p* < 0.001, *p* < 0.01, *p* < 0.05, respectively; ns = not significant.

**Table 4:** Average pod weight (g), pod number (number plant^−1^) and total fresh-pod-yield (g plant^−1^) of the common bean (*Phaseolus vulgaris* L.) in response to the varying quantities of inorganic-N application (100%, 75–25, or 50–25%), and/or inoculation with rhizobia strains (PVTN21 or CIAT899).

Treatments		Yield Components		
Nitrogen	Strain	Pod Yield	Pod Number	Pod Size
		(g Plant^−1^)	(N^o.^ Plant^−1^)	(g)
Main effects				
100%		276	17.3	15.95
75–25%		155	10.5	14.78
50–25%		214	13.9	15.41
	Non-inoculated	195	13.0	14.86
	PVTN21	233	14.7	15.75
	CIAT 899	217	13.9	15.55
Interactions				
100%	Non-inoculated	273 a	17.2 a	15.87 ab
	PVTN21	282 a	17.4 a	16.20 a
	CIAT 899	275 a	17.4 a	15.78 ab
75–25%	Non inoc.	133 e	9.4 f	14.12 e
	PVTN21	172 d	11.2 de	15.35 bc
	CIAT 899	161 d	10.8 e	14.88 cd
50–25%	Non-inoculated	180 d	12.4 cd	14.58 de
	PVTN21	245 b	15.6 b	15.68 ab
	CIAT 899	217 c	13.6 c	15.97 ab
Statistical Significance				
Nitrogen		***	***	***
Strain		***	***	***
Nitrogen * Strain		*	*	*

Mean values (*n* = 6) followed by different letters within the same column indicate significant differences according to Duncan’s multiple range test (*p* < 0.05: *** and * significant at *p* < 0.001, *p* <0.01 and *p* < 0.05, respectively.

**Table 5:** Chemical composition of the applied NS.

NS		100% N	75% N	50% N	25% N	100% N	25% N
DACE *		0–50	0–35	0–35	35–50	50–80	50–80
pH		5.50	5.50	5.50	5.50	5.50	5.50
EC (dS/m)		1.93	1.93	1.93	1.69	1.90	1.78
Κ	mM	5.3	5.3	5.3	5.3	5	5.3
Ca	mM	3.75	3.75	3.75	3.01	3.55	3.01
Mg	mM	1.6	1.6	1.6	1.6	1.5	1.6
NO_3_	mM	12.6	9.15	5.7	3.135	12	2.3
NH_4_	mM	1.2	1.2	1.2	0.315	1.8	1.15
H_2_PO_4_	mM	1.2	1.2	1.2	1.2	1.2	1.2
SO_4_	mM	3.07	5.02	8.47	8.27	3.37	9.3
Cl	mM	0.3	1.8	1.8	2.2	0.3	2.84
Fe	μM	15	15	15	15	15	15
Mn	μM	7	7	7	7	7	7
Zn	μM	5	5	5	5	5	5
Cu	μM	0.7	0.7	0.7	0.7	0.7	0.7
B	μM	20	20	20	20	20	20
Mo	μM	0.5	0.5	0.5	0.5	0.5	0.5

* DACE denotes days after crop establishment. EC denotes electrical conductivity.

## Data Availability

Not applicable.

## Supplementary Materials

The following supporting information can be downloaded at: https://www.mdpi.com/article/10.3390/plants12030646/s1, Table S1: Chemical composition of irrigation water, Table S2: Nutrient solution recipes.
