# Supplementary material for: Hydroponic Common-Bean Performance under Reduced N-Supply Level and Rhizobia Application
Source: Plants (Basel). 2023 Feb 1;12(3):646. doi: 10.3390/plants12030646 (PMC9920343; doi:10.3390/plants12030646)
Supplement: Supplementary file 1 [file plants-12-00646-s001.zip › plants-2140643-supplementary.pdf]

Table S1. Chemical composition of irrigation water.

Parameter	Value
EC	0.315 ds/m
pH	7.3
Ca^{2+}	1.00 mmol/l
Mg^{2+}	0.30 mmol/l
K^{+}	0.00 mmol/l
NH^{4+}	0.00 mmol/l
Na^{+}	0.30 mmol/l
$SO4^{2-}$	0.20 mmol/l
NO^{3-}	0.00 mmol/l
$H2PO^{4-}$	0.00 mmol/l
HCO^{3-}	2.20 mmol/l
Cl^{-}	0.30 mmol/l
Fe	0.00 μmol/l
Mn^{++}	0.00 μmol/l
Zn^{++}	2.15 mmol/l
Cu^{++}	0.00 μmol/l
B	0.00 μmol/l
Mo	0.00 μmol/l
Si	0.00 μmol/l

Table S2. Nutrient solution recipes

Information about concentrated stock solutions								
**Tanks (n=3)**	**Volume (L)**		**Concentration factor**					
Tank 1	20 L		100					
Tank 2	20 L		100					
Nitric acid	20 L		100					
	NS		100% N	75% N	50% N	25% N	100% N	25% N
	DACE Applied		0-50	0-35	0-35	35-50	50-80	50-80
**Tank 1 Fertilizers**	Calcium nitrate		1.189 kg	1.189 kg	1.189 kg	0.677 kg	1.102 kg	0.320 kg
	Calcium chloride (48% Cl)		0.000 kg	0.333 kg	0.333 kg	0.422 kg	0.000 kg	0.564 kg
	Potassium nitrate		0.288 kg	0.261 kg	0.000 kg	0.006 kg	0.218 kg	0.000 kg
	Ammonium nitrate		0.104 kg	0.104 kg	0.104 kg	0.000 kg	0.207 kg	0.000 kg
	Fe- EDDHA (13% Fe)		13.00 g	13.00 g	13.00 g	13.00 g	13.00 g	13.00 g
**Tank 2 Fertilizers**	Potassium nitrate		0.770 kg	0.403 kg	0.000 kg	0.000 kg	0.678 kg	0.000 kg
	Magnesium sulphate		0.640 kg	0.640 kg	0.640 kg	0.640 kg	0.591 kg	0.640 kg
	Magnesium nitrate		0.000 kg	0.000 kg	0.000 kg	0.000 kg	0.000 kg	0.000 kg
	Monopotassium phosphate		0.000 kg	0.000 kg	0.000 kg	0.000 kg	0.000 kg	0.000 kg
	Potassium sulphate		0.012 kg	0.352 kg	0.924 kg	0.918 kg	0.099 kg	0.924 kg
	Phosphoric acid (85%)		0.164 L	0.164 L	0.164 L	0.164 L	0.164 L	0.164 L
	Ammonium sulphate		0.000 kg	0.000 kg	0.000 kg	0.000 kg	0.000 kg	0.264 kg
	Manganese sulphate		2.37 g	2.37 g	2.37 g	2.37 g	2.37 g	2.37 g
	Zinc sulphate		1.64 g	1.64 g	1.64 g	1.64 g	1.64 g	1.64 g
	Copper sulphate		0.35 g	0.35 g	0.35 g	0.35 g	0.35 g	0.35 g
	Boric acid (17% B)		2.47 g	2.47 g	2.47 g	2.47 g	2.47 g	2.47 g
	Sodium molybdate		0.24 g	0.24 g	0.24 g	0.24 g	0.24 g	0.24 g
**Tank 3**	Nitric acid		0.174 L	0.174 L	0.174 L	0.174 L	0.174 L	0.174 L

The above recipes were scheduled based on the chemical composition of irrigation water (Table S1).
